# Supplementary material for: Poking COVID-19: Insights on Genomic Constraints among Immune-Related Genes between Qatari and Italian Populations
Source: Genes (Basel). 2021 Nov 22;12(11):1842. doi: 10.3390/genes12111842 (PMC8623290; doi:10.3390/genes12111842)
Supplement: Supplementary file 1 [file genes-12-01842-s001.zip › Figure S1.pdf]

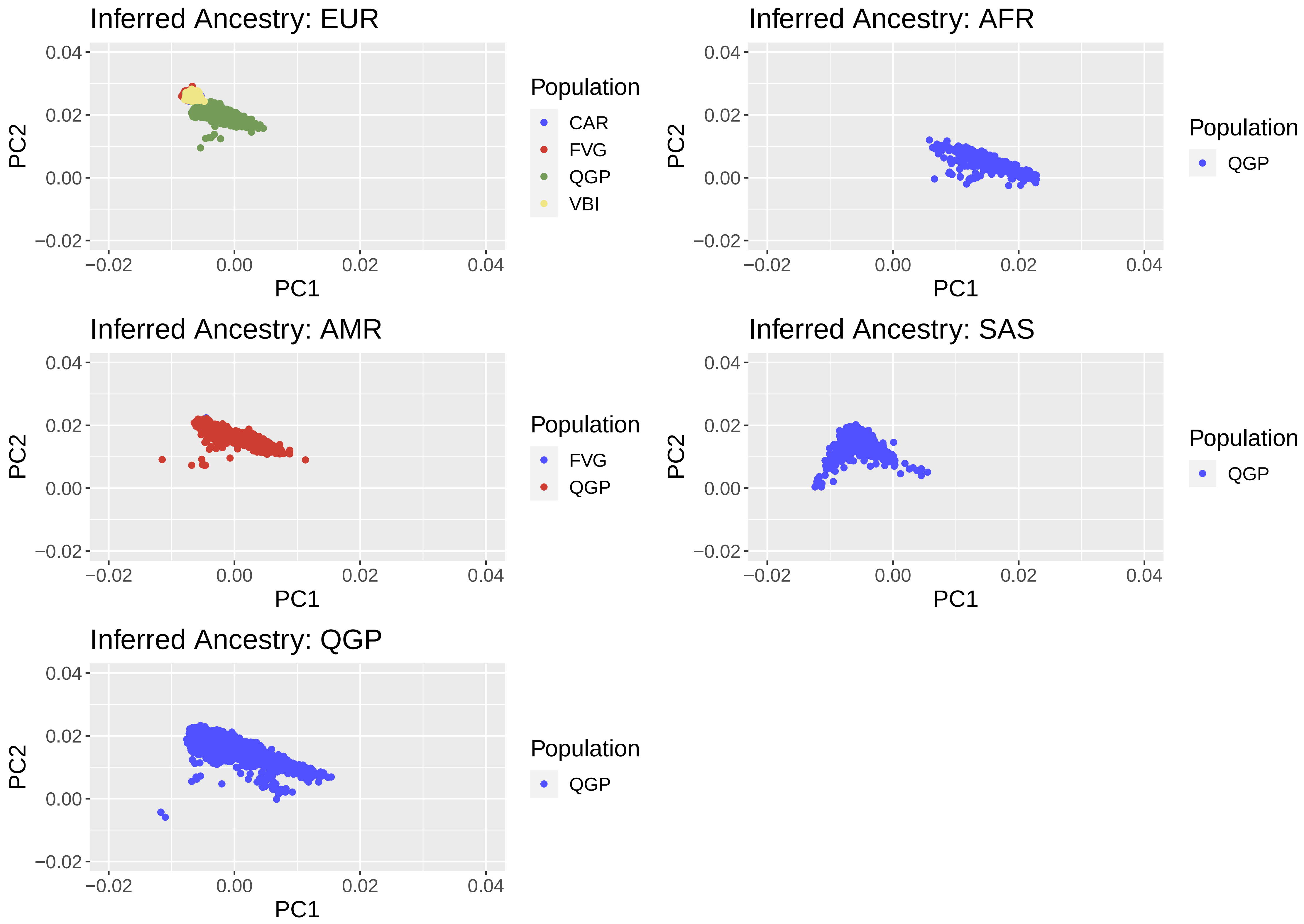
Inferred Ancestry: EUR
PC2
PC1
0.04
0.02
0.00
−0.02
−0.02
0.00
0.02
0.04
Population
CAR
FVG
QGP
VBI
Inferred Ancestry: AFR
PC2
PC1
0.04
0.02
0.00
−0.02
−0.02
0.00
0.02
0.04
Population
QGP
Inferred Ancestry: AMR
PC2
PC1
0.04
0.02
0.00
−0.02
−0.02
0.00
0.02
0.04
Population
FVG
QGP
Inferred Ancestry: SAS
PC2
PC1
0.04
0.02
0.00
−0.02
−0.02
0.00
0.02
0.04
Population
QGP
Inferred Ancestry: QGP
PC2
PC1
0.04
0.02
0.00
−0.02
−0.02
0.00
0.02
0.04
Population
QGP
