# Supplementary material for: Poking COVID-19: Insights on Genomic Constraints among Immune-Related Genes between Qatari and Italian Populations
Source: Genes (Basel). 2021 Nov 22;12(11):1842. doi: 10.3390/genes12111842 (PMC8623290; doi:10.3390/genes12111842)
Supplement: Supplementary file 1 [file genes-12-01842-s001.zip › supplementary_tables.pdf]

Table S2: List of genes recently associated with COVID-19 susceptibility and severity.

Gene	Variants	Source	Publication
SLC6A2	rs35081325	HGI	not published in a peer review journal
CXCR6		HGI	
CCR1		HGI	
OAS1	rs4766664	HGI	
OAS2		HGI	
OAS3		HGI	
DPP9	rs2277732	HGI	
TYK2	rs7956615	HGI	
IFNAR2	rs13050728	HGI	
IL10RB		HGI	
IFNAR1		HGI	
IFNAR2		HGI	
FOXP4	rs1853837	HGI	
TLR3		Human Genetic Effort (HGE)	Zhnag et al, Science 2020
UNC93B1		Human Genetic Effort (HGE)	
TICAM1		Human Genetic Effort (HGE)	
TBK1		Human Genetic Effort (HGE)	
IRF3		Human Genetic Effort (HGE)	
IRF7		Human Genetic Effort (HGE)	
LZTFL1		Genomicc UK	Castineira et al MedriXiv
CCHCR1		Genomicc UK	
HLA-DPB1		Genomicc UK	
VSTM2A		Genomicc UK	
HAS2-AS1		Genomicc UK	
IGF1		Genomicc UK	
ABO			

Table S3: Details of the comparisons defined between Target and Reference populations.

		Target 1	Reference 1		Target 2	Reference 2
**Population specific**	**C1**	Relaxed (+)	Costrained (-)	vs	Costrained (-)	Relaxed (+)
	**C2**	Costrained (-)	Relaxed (+)	vs	Relaxed (+)	Costrained (-)
**Population specific vs ancestry related**	**C3**	Relaxed (+)	Costrained (-)	vs	Costrained (-)	Costrained (-)
	**C4**	Costrained (-)	Relaxed (+)	vs	Relaxed (+)	Relaxed (+)
	**C5**	Costrained (-)	Costrained (-)	vs	Relaxed (+)	Costrained (-)
	**C6**	Relaxed (+)	Relaxed (+)	vs	Costrained (-)	Relaxed (+)
**Ancestry related**	**C7**	Relaxed (+)	Relaxed (+)	vs	Costrained (-)	Costrained (-)
	**C8**	Costrained (-)	Costrained (-)	vs	Relaxed (+)	Relaxed (+)

Table S 4: Summary statistics of the phenotype information availabe for the TOV cohort.

		Gender		Outcome		
**Disease Severity group**	**n**	**M (n)**	**F (n)**	**Survived (n)**	**Deceased (n)**	**Age (mean)**
Asymptomatic/Paucisymptomatic (1)	52	25	27	52	0	37.5
Severe (2)	73	45	28	60	13	60.4
Critical/life-threatening (3)	64	40	24	21	43	68.4
**total**	189	110	79	133	56	57.73

Table S5: Comparison between scores distribution of target populations versus reference populations.

Reference	Target	P-value DSC	P-value SSC
**EUR**	**CAR**	1.33E-06	7.79E-07
**EUR**	**FVG**	3.17E-02	3.65E-08
**EUR**	**VBI**	3.78E-03	3.84E-07
**AFR**	**QGP**	4.64E-07	4.36E-01
**SAS**	**QGP**	2.97E-05	5.35E-01

Table S6: Enrichment tests in target populations versus reference populations.

		DSC score		SSC score	
**Reference**	**Target**	**Costrained p-value**	**Relaxed p-value**	**Costrained p-value**	**Relaxed p-value**
**EUR**	**CAR**	2.08E-06	6.81E-04	1.38E-01	7.11E-04
**EUR**	**FVG**	2.27E-03	7.70E-02	3.19E-01	7.80E-06
**EUR**	**VBI**	2.27E-03	1.93E-03	2.82E-01	3.68E-06
**AFR**	**QGP**	3.72E-03	4.17E-01	1.00E+00	8.60E-01
**SAS**	**QGP**	3.51E-02	2.52E-01	7.51E-01	8.60E-01

Table S7: DSC and SSC scores for *FLNA*, *MUC5AC* and *ABCA7* in all 1000Genomes superpopulations.

Gene Name	Score	AFR	AMR	EAS	EUR	SAS
ABCA7	**DSC**	2.6813	1.6151	2.5922	3.0203	2.1504
	**SSC**	3.3248	4.0146	3.7889	2.5907	3.9976
FLNA	**DSC**	-2.1657	-2.0498	-2.2511	-2.3994	-1.8791
	**SSC**	-3.3509	-3.3736	-3.2086	-3.0729	-3.0966
MUC5AC	**DSC**	3.4995	2.8133	2.8882	3.4774	3.0323
	**SSC**	3.2673	4.0639	4.1692	3.6789	4.2443

Table S8: Comparison of Gene Singleton Density between the Covid-19 positive cohort (TOV) and other target and reference populations. All singleton counts have been adjusted considering the sample size of each cohort.

Transcript ID	Gene name	Cohort	P-value	OR	TOV Gene singleton count	TOV Gene singleton density	Cohort Gene singleton count	Cohort Gene singleton density
**ENST00000369850**	***FLNA***	**CAR**	0.630140	1.4201	13	0.00050	6	0.00023
		**FVG**	0.046901	2.3537	13	0.00050	11	0.00042
		**VBI**	0.000458	5.8145	13	0.00050	5	0.00019
		**QGP**	0.000028	4.3237	13	0.00050	229	0.00877
		**EUR**	0.312323	0.6893	13	0.00050	61	0.00234
		**AFR**	0.878006	0.9218	13	0.00050	60	0.00230
		**SAS**	0.200408	0.6464	13	0.00050	61	0.00234
**ENST00000263094**	***ABCA7***	**CAR**	3.2746E-11	10.9752	84	0.00328	5	0.00020
		**FVG**	3.1278E-23	12.8380	84	0.00328	13	0.00051
		**VBI**	7.1607E-21	8.5370	84	0.00328	22	0.00086
		**QGP**	6.2413E-63	17.8135	84	0.00328	359	0.01404
		**EUR**	4.4467E-10	2.9802	84	0.00328	91	0.00356
		**AFR**	1.7360E-08	2.5141	84	0.00328	142	0.00555

		**SAS**	2.3435E-04	1.8442	84	0.00328	138	0.00540
		**CAR**	7.4274E-12	7.4508	114	0.00264	10	0.00023
		**FVG**	1.8836E-31	15.0962	114	0.00264	15	0.00035
		**VBI**	2.3148E-36	19.6004	114	0.00264	13	0.00030
**ENST00000621226**	***MUC5AC***	**QGP**	1.5512E-76	15.4443	114	0.00264	562	0.01301
		**EUR**	3.3142E-06	1.9908	114	0.00264	185	0.00428
		**AFR**	2.9701E-08	2.2129	114	0.00264	219	0.00507
		**SAS**	7.2316E-03	1.4702	114	0.00264	235	0.00544

Table S9: Comparison of Coding Singleton Density between the Covid-19 positive cohort (TOV) and other target and reference populations. All singleton counts have been adjusted considering the sample size of each cohort.

Transcript ID	Gene name	Cohort	P-value	OR	TOV CDS singleton count	TOV CDS singleton density	Cohort CDS singleton count	Cohort CDS singleton density
		**CAR**	0.409653	3.2705	5	0.00063	1	0.00013
		**FVG**	0.316565	1.9922	5	0.00063	5	0.00063
		**VBI**	0.013015	11.1693	5	0.00063	1	0.00013
**ENST00000369850**	***FLNA***	**QGP**	0.039803	2.7799	5	0.00063	137	0.01725
		**EUR**	0.786342	1.1543	5	0.00063	14	0.00176
		**AFR**	0.787767	1.1814	5	0.00063	18	0.00227
		**SAS**	0.813561	0.7582	5	0.00063	20	0.00252
		**CAR**	2.5959E-07	14.3697	44	0.00683	2	0.00031
		**FVG**	1.0535E-17	43.6709	44	0.00683	2	0.00031
		**VBI**	2.0060E-16	19.6584	44	0.00683	5	0.00078
**ENST00000263094**	***ABCA7***	**QGP**	1.5606E-40	24.9877	44	0.00683	134	0.02080
		**EUR**	1.0966E-08	4.0565	44	0.00683	35	0.00543
		**AFR**	5.3713E-09	3.8931	44	0.00683	48	0.00745
		**SAS**	3.7924E-06	2.9601	44	0.00683	45	0.00699
		**CAR**	8.4692E-11	12.4070	76	0.00448	4	0.00024
		**FVG**	2.7623E-24	18.8715	76	0.00448	8	0.00047
		**VBI**	3.2118E-30	42.4223	76	0.00448	4	0.00024
**ENST00000621226**	***MUC5AC***	**QGP**	1.2101E-64	23.1499	76	0.00448	250	0.01474
		**EUR**	4.0071E-08	2.6675	76	0.00448	92	0.00542
		**AFR**	1.0241E-10	3.1351	76	0.00448	103	0.00607
		**SAS**	9.9471E-05	1.9678	76	0.00448	117	0.00690

Table S10: Multinomial analysis results, comparing classes of disease severity, considering the burden of singletons in the whole gene. Class 1: Asymptomatic/Paucisymptomatic; Class 2: Severe; Class 3: Critical/life-threatening. Only significant predictors are reported.

						IC 95%		
**Severity Class**	**Predictor**	**Estimate**	**Std. Error**	**P-value**	**OR**	**Lower bound**	**Upper bound**	**Std. Error (OR)**
**Class 2 vs Class 1**	**Age**	6.72E-02	1.36E-02	8.35E-07	1.0695029	1.0412933	1.0984768	0.0145859
	**Gender (Male)**	9.25E-01	4.63E-01	0.05	2.5208194	1.0173269	6.2463014	1.1670436
**Class 3 vs Class 1**	**Age**	1.01E-01	1.63E-02	4.92E-10	1.1064869	1.0717711	1.1423270	0.0179959
	**Gender (Male)**	1.10E+00	5.09E-01	0.03	2.9987634	1.1061065	8.1299424	1.5259327
**Class 3 vs Class 2**	**Age**	3.40E-02	1.17E-02	0	1.0345794	1.0111462	1.0585557	0.0120932

Table S11: Multinomial analysis results, comparing classes of disease severity, considering the burden of singletons in the coding regions of each gene. Class 1: Asymptomatic/Paucisymptomatic; Class 2: Severe; Class 3: Critical/life-threatening. Only significant predictors are reported.

						IC 95%		
**Severity Class**	**Predictor**	**Estimate**	**Std. Error**	**P-value**	**OR**	**Lower bound**	**Upper bound**	**Std. Error (OR)**
**Class 2 vs Class 1**	**Age**	6.87E-02	1.38E-02	6.09E-07	1.0711545	1.0426111	1.1004793	0.0147605
	**Gender (Male)**	9.39E-01	4.68E-01	0.04	2.5573716	1.0227587	6.3946161	1.1958014
	FLNA	-1.04E+01	7.39E-05	< 2.2e-16	0.0000296	0.0000296	0.0000296	0.0000000022
**Class 3 vs Class 1**	**Age**	9.86E-02	1.60E-02	6.42E-10	1.1036457	1.0696605	1.1387107	0.0176120
	**Gender (Male)**	1.18E+00	5.08E-01	0.02	3.2520969	1.2007352	8.8080493	1.6532035
**Class 3 vs Class 2**	**Age**	2.99E-02	1.14E-02	0.01	1.0303340	1.0076149	1.0535653	0.0117211
	FLNA	7.98E+00	5.18E-01	< 2.2e-16	2933.6216596	1063.3832398	8093.1650220	1518.8708798

Table S12: Logistic analysis results for disease outcome classification. Only significant predictors are reported.

						IC 95%		
**Outcome**	**Predictor**	**Estimate**	**Std. Error**	**P-value**	**OR**	**Lower bound**	**Upper bound**	**Std. Error (OR)**
**Survied vs Deceased**	**Age**	1.07E-01	1.67E-02	1.50E-10	1.1129343	1.0770954	1.1499656	0.0185860
	**ABCA7**	4.20E-01	1.85E-01	0.0228	1.5225705	1.0603341	2.1863118	0.2810665

Table S13: Logistic analysis results for disease outcome classification considering the burden of singletons in the coding regions of each gene. Only significant predictors are reported.

						IC 95%		
**Outcome**	**Predictor**	**Estimate**	**Std. Error**	**P-value**	**OR**	**Lower bound**	**Upper bound**	**Std. Error (OR)**
**Survied vs Deceased**	**Age**	1.05E-01	1.66E-02	2.33E-10	1.1110439	1.0754556	1.1478098	0.0184544
