# Supplementary figures and images for: Poking COVID-19: Insights on Genomic Constraints among Immune-Related Genes between Qatari and Italian Populations
Source: Genes (Basel). 2021 Nov 22;12(11):1842. doi: 10.3390/genes12111842 (PMC8623290; doi:10.3390/genes12111842)

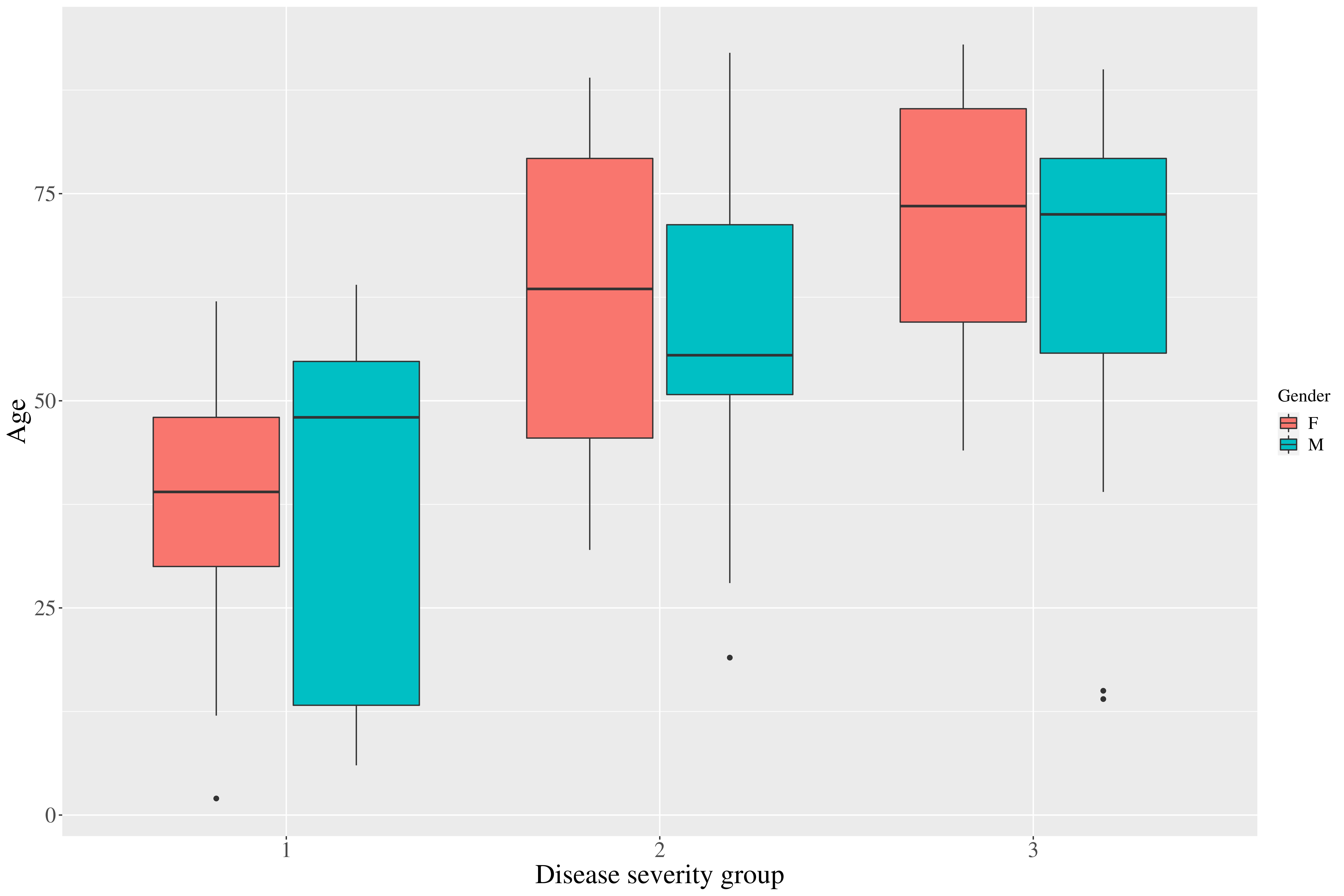

Supplement: Supplementary file 1 [file genes-12-01842-s001.zip › Figure S2.jpeg]

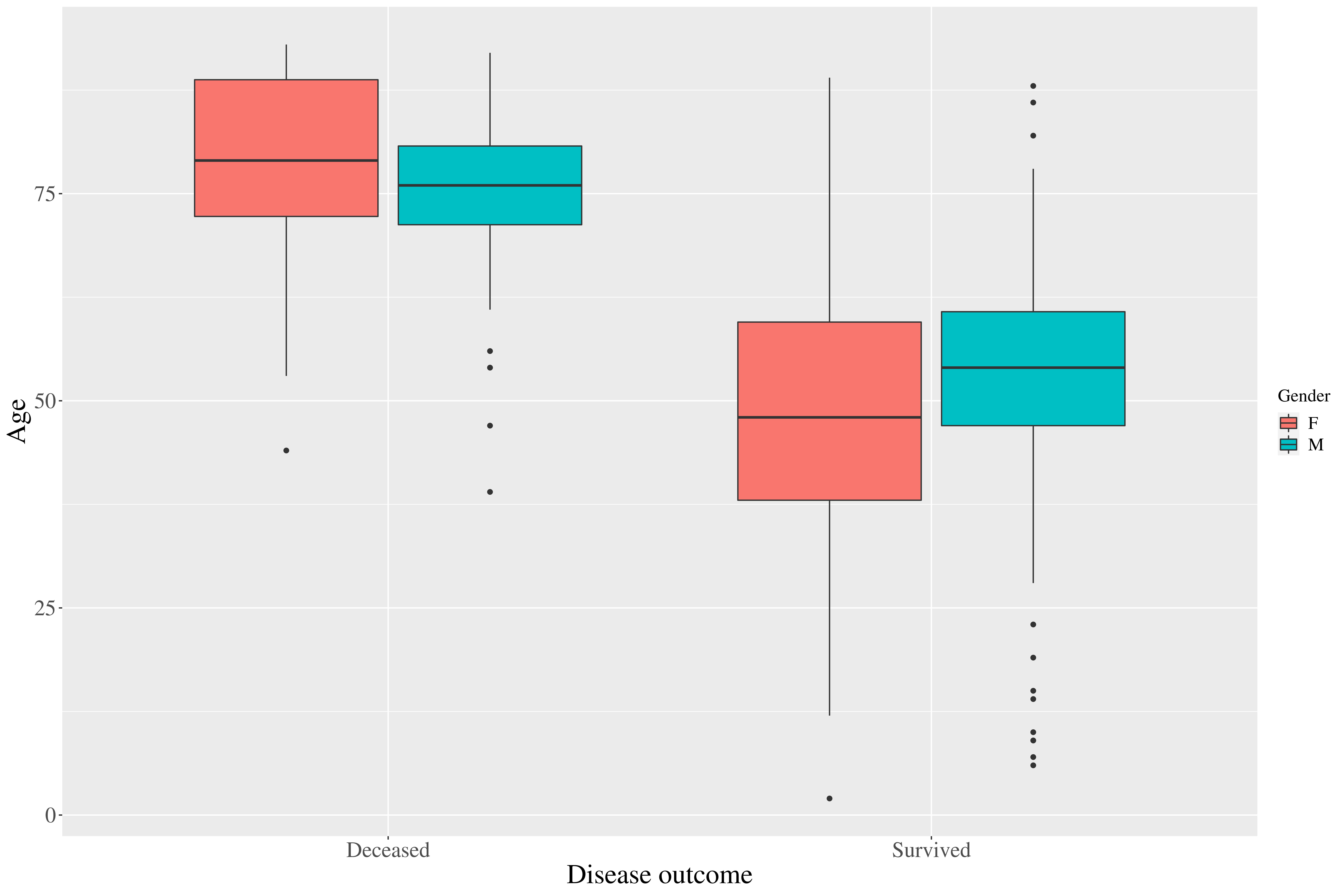

Supplement: Supplementary file 1 [file genes-12-01842-s001.zip › Figure S3.jpeg]
